# Investigation of the long-term prognosis (3–13 Years) of full mouth implant prosthetics combining zygomatic implants based on the all-on-four concept

**DOI:** 10.1186/s40729-026-00700-7

**Published:** 2026-07-03

**Authors:** Takashi Uesugi, Yoshiaki Shimoo, Motohiro Munakata, Daisuke Sato, Kikue Yamaguchi, Minoru Sanda, Michiya Fujimaki, Kazuhisa Nakayama, Tae Watanabe, Paulo Malo

**Affiliations:** 1 Department of Implant Dentistry, Showa Medical University School of Dentistry, 2-1-1, Kita-senzoku, Ota-ku, Tokyo, 145-8515 Japan; 2 Malo Dental & Medical Tokyo, Fukuhara Ginza 8F, 7-8-10 Ginza, Chuo-ku, Tokyo, 104- 0061 Japan; 3 Department of Prosthodontics, Showa Medical University School of Dentistry, 2-1-1, Kita- senzoku, Ota-ku, Tokyo, 145-8515 Japan

**Keywords:** All-on-four, Zygomatic implants, Implant failure, Complete edentulous, Systemic disease

## Abstract

**Purpose:**

Zygomatic implants (ZIs) are indicated for severely atrophic maxillae where conventional implant (CI) placement is difficult. Although ZIs show favourable outcomes, factors influencing their survival remain unclear. This retrospective study evaluated implant- and patient-related factors associated with ZI survival in full-arch immediate-loading rehabilitation using the all-on-four concept combining ZIs and CIs.

**Methods:**

A total of 923 implants (323 ZIs and 600 CIs) placed in 203 patients between 2010 and 2021 were analysed. Cumulative survival rates were estimated using the Kaplan–Meier method, and intergroup comparisons were performed using the log-rank test. Cox proportional hazards regression analyses were performed to calculate hazard ratios (HRs). Statistical significance was set at *p* < 0.05.

**Results:**

Cumulative survival rates at 3–13 years were 94.5% and 95.9% at the patient and implant levels, respectively, for ZIs, and 97.9% and 98.7%, respectively, for CIs. ZIs showed significantly lower implant-level survival than CIs (*p* = 0.0178). Palatal positioning of the ZI platform was significantly associated with reduced implant-level survival (HR = 18.177, 95% CI: 1.418–233.053, *p* = 0.026). Systemic disease was significantly associated with reduced patient-level ZI survival after adjustment for sex and smoking status (HR = 14.872, 95% CI: 1.812–122.077, *p* = 0.012).

**Conclusions:**

Immediate full-arch rehabilitation combining ZIs and CIs achieved high long-term survival; however, palatal platform positioning and systemic disease were associated with ZI failure.

## Background

The number of completely edentulous patients is increasing worldwide, having increased two-fold over the past 30 years. Aging of the global population shifted the average age of becoming completely edentulous from 70 to 74 years of age in 1990, to 75–79 years in 2021. Moreover, the global overall percentage of completely edentulous patients is predicted to increase from 4,773 cases/100,000 people in 2021 to 5,004 cases/100,000 people in 2040 [1]. Loss of teeth not only deteriorates oral function but is also associated with various systemic diseases, such as cardiovascular disease and dementia [2, 3].

Maló proposed the all-on-four concept, which involves immediate evading bone augmentation for completely edentulous patients or patients with terminal dentition. In 2005, Maló presented a case for which only conventional implants (CIs) were used in the maxilla [4]. Later in 2008, Maló described a treatment combining zygomatic implants (ZIs) for a case that could not be treated with tilted CIs due to pneumatisation of the maxillary sinus extending to the canine region [5].

ZIs were clinically introduced in 1989 by Bränemark, and their treatment outcomes were first presented in 1993 [6]. ZIs were primarily used to provide stability to the prosthesis in patients with complete or partial loss of the maxilla owing to conditions such as tumour resection. Bone grafts were subsequently employed in combination, with delayed loading. With advancements in the surgical techniques, implant design, and surface shape, ZIs were later adopted for treatments, even with immediate loading [7, 8]. Currently, they are widely applied for cases of severe atrophy, those of recovery for failed implant (and/or bone graft), and those in which bone grafting should be avoided owing to the presence of systemic diseases [9].

In a systematic review of ZIs, the mean ZI survival rate for 36 to 141.6 months (estimated mean survival: 75.4 months) was 96.2% when including delayed-loaded situations, and 98.1% for immediately loaded situation, (estimated mean survival: 73.6 months), indicating a favourable prognosis [10]. For treatment based on the all-on-four concept combining ZIs, the cumulative patient-level survival rates of 6–84.2 months (estimated mean survival: 83.2 months) were 98.2% and 96.7% for ZIs and CIs, respectively, indicating a favourable prognosis [11]. Thus, the use of ZIs, which have been established as a reliable long-term treatment option for immediate loading, has been increasing dramatically.

In a previous study, we reported that the long-term prognosis for maxillary treatment based on the all-on-four concept using only CIs was favourable in Japanese patients, with a patient-level survival rate of 94.4% and an implant-level survival rate of 97.4% (estimated mean survival: 105.7 months) [12]. However, no studies have reported on the long-term prognosis of the all-on-four concept with ZIs in a Japanese population.

Furthermore, ZIs tend to be evaluated in the same way as CIs placed in the alveolar bone. However, as the maxilla in which ZIs are indicated is characterised by severe atrophy of both the alveolar and basal bone and is primarily anchored to the zygomatic bone, the biomechanical environment differs significantly from that of CIs. As complications also differ, separate evaluation criteria are used for ZIs regarding success and survival [13], and it is anticipated that the risk factors associated with survival rates will also differ from those for CIs. Although male sex has been reported as a risk factor impacting ZIs survival rates [14], no other patient- or implant-related risk factors have been identified, and this topic remains insufficiently investigated.

The hypothesis of this study was the existence of specific risk factors influencing the survival rates of ZIs.Therefore, this study investigated the 3–13 year cumulative survival rates for treatment using the all-on-four concept combining ZIs and CIs and examined the risk factors affecting survival rates.

## Methods

### Study population and ethics

Study participants comprised cases of edentulous maxilla which are the indication for the all-on-four concept, with CIs implanted in the anterior region and ZIs implanted on one or both sides of the posterior regions (for cases in which ZIs were only implanted on one side, tilted CIs were inserted on the other side). All participants were treated at a private clinic (MALO Dental & Medical TOKYO, Tokyo, Japan) between March 2010 and March 2021. In all cases, a preoperative general medical examination was performed to confirm that the patient was in a suitable condition for surgery before commencing treatment.

Exclusion criteria.

- ASA score ≥ 3 or other systemic contraindications to surgery.
- Patients with a history of diabetes whose HbA1c is not controlled at 6.9% or below
- Alcohol or drug abuse.
- Pregnancy or lactation.

In all cases, preoperative examinations were performed using CBCT (TrophyMax, Trophy Radiologie, Vincennes, France). When sinusitis was identified, appropriate treatment for sinusitis was provided prior to surgery.

The exclusion criteria were patients who did not undergo follow-up.

The study was conducted in accordance with the Declaration of Helsinki, and the Ethics Committee of Showa Medical University approved the study protocol (ethics review committee number 11000688 approval, approval number: 21-055-A; approval date: 24 September 2021); all the patients provided written informed consent for participation in the study. This study was reported in accordance with the STROBE reporting guideline [15].

### Surgical protocol

**Fig. 1:**
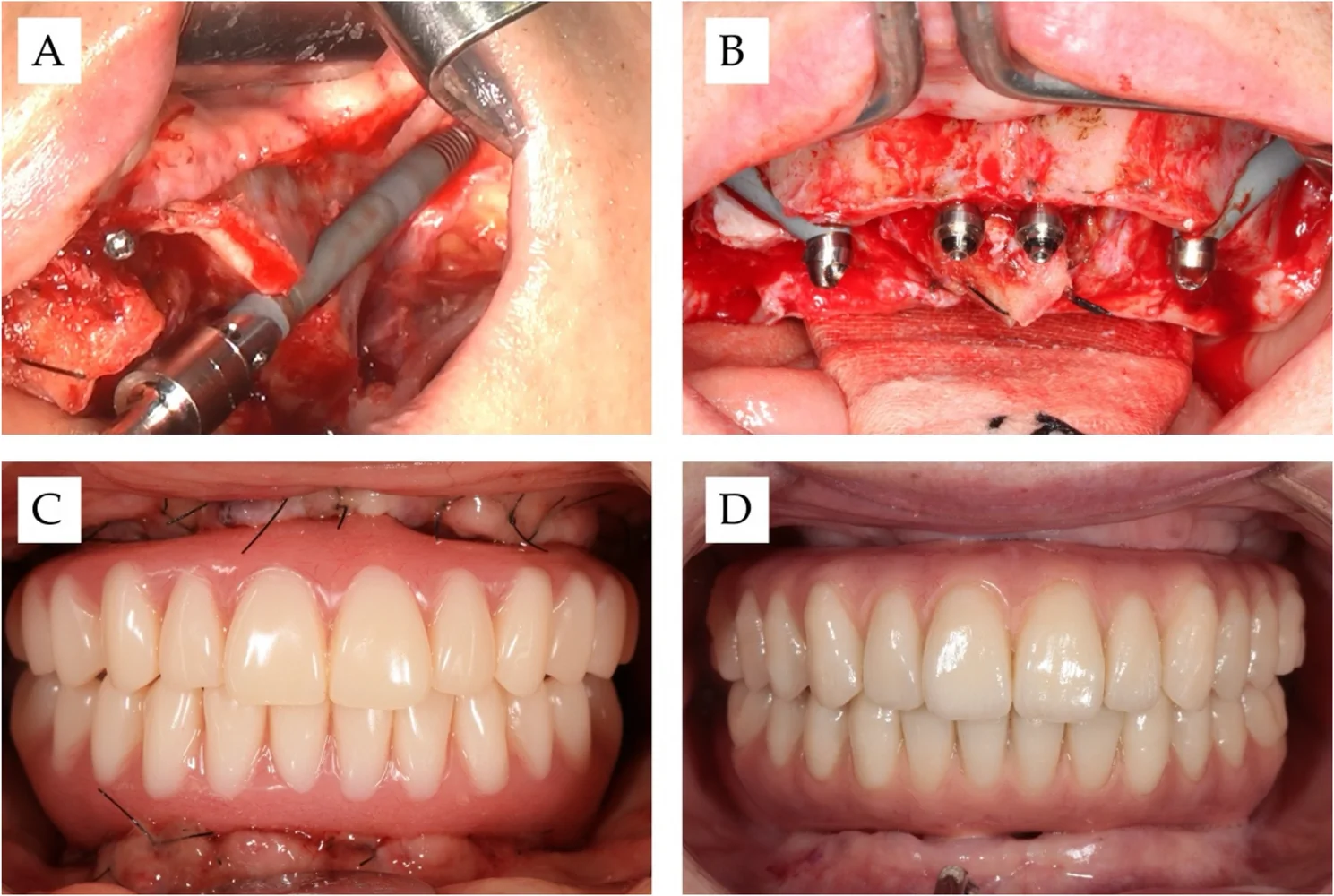
Surgical and prosthetic protocol. **A** Insertion of the zygomatic implants. **B** Placement of the implants and abutments. **C** Immediate loading with provisional restoration after surgery. **D** Final prosthesis

**Fig. 2:**
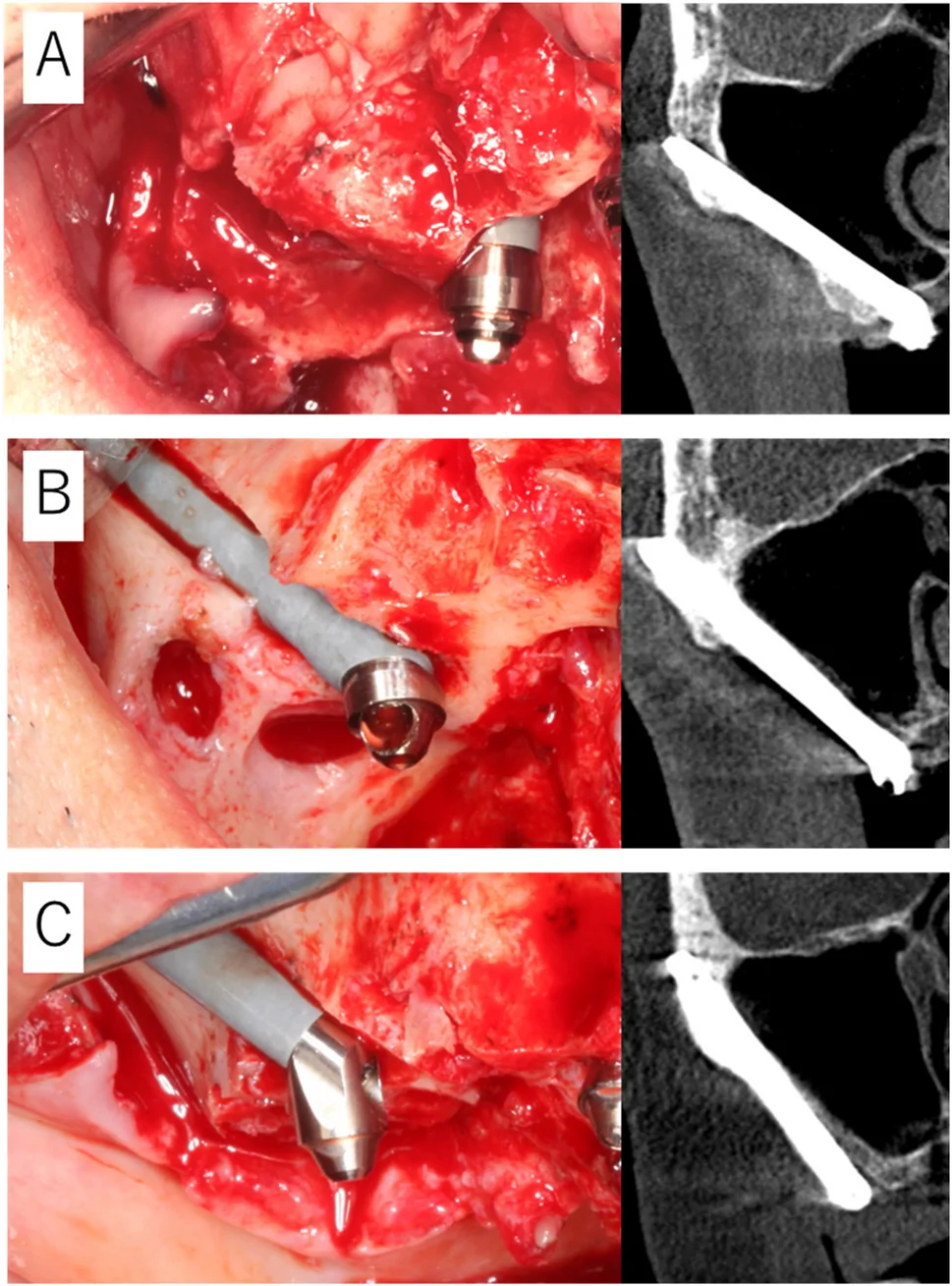
Position of the zygomatic implant’s platform. **A** Palatal. **B** Ridge. **C** Vestibular

Surgery was performed under general anaesthesia or intravenous sedation with local anaesthesia (2% lidocaine containing 1/80,000 adrenaline). The surgical and prosthetic protocol was as follows (Fig. 1):

1. For patients with remaining teeth, all the remaining teeth in maxilla were extracted.
2. A longitudinal incision was made in the mucosa distal to the first molars on both sides, and a transverse incision was made in the alveolar crestal mucosa at the lingual/palatal side to raise the mucoperiosteal flap.
3. When needed, removal of the top of the alveolar crest was performed to provide adequate prosthetic space and flattened mucosal surface to stimulate clensability under the prosthesis.
4. Anterior implants with a diameter of ≥ 3.3 mm were placed in the area corresponding to the central and lateral incisors.
5. For the posterior sites, implants with a diameter of ≥ 4.0 mm were used. The implant position was determined using a standardised surgical guide (All-on-four Guide, Nobel Biocare AG, Kloten, Switzerland). The placement of ZIs was performed by the same experienced specialist. The ZI placement protocol was based on the concept of the anatomy-guided approach (AGA). The position of the ZI platform was determined preoperatively based on CBCT images, considering the morphology and bone quality of the alveolar bone, maxilla, and zygomatic bone, as well as the design of the planned prosthetic restoration, and was classified into three categories: palatal, ridge, and vestibular (position of the ZI’s platform; Fig. 2). First, a small bony window was created in the maxillary wall, and the sinus mucosa was elevated as required to improve drilling direction visibility from the alveolar bone to the zygomatic bone. After preparing the implant site, the ZIs were placed, the platform height was adjusted according to the maxillary alveolar bone crest, and the ZIs were inserted to the zygomatic bone. In this study, computer-assisted implant surgery was not performed in any of the cases.

6. Four implants per patient were initially placed When CIs with a length of ≥ 10 mm could not be placed, or if an insertion torque of any of the implants were below 35 Ncm, additional CIs were placed nearby if surgically feasible.
7. After implant placement, abutments (Multi-unit Abutment, Nobel Biocare AG, Kloten, Switzerland) were connected (tightening torque: 35 Ncm for straight, 45° and 60°, 15 Ncm for 17° and 30°) and the flaps were sutured.

To prevent postoperative infection, patients received 250 mg of amoxicillin four times daily for 5 days and 0.2% benzethonium chloride mouthwash four times a day for 2 weeks, as well as 60 mg of loxoprofen sodium three times a day for 5 days as an analgesic. Sutures were removed at 2 weeks postoperatively.

### Prosthetic protocol

Briefly, impression copings for open tray impression technique in each abutment were connected, then impression and bite registration were taken. Using the indirect method, a provisional restoration was fabricated with embedded reinforcing framework made of a Co-Cr alloy into a titanium temporary cylinder and acrylic resin (PROVISTA, Sun Medical Co., Ltd., Shiga, Japan). This provisional restoration was delivered at the same day.

The patients were instructed to maintain a soft diet for 2 − 3 months postoperatively. Follow-up clinical appointments were performed at 7 and 14 days, and every month during the subsequent 6 months.

The final prosthesis was fabricated 6 months after the surgery, using a titanium framework with ceramic crowns (IPS e.max Press, Ivoclar Vivadent, Schaan, Liechtenstein) or with acrylic resin crowns (anterior teeth [Bioblend, Dentsply Sirona, Bensheim, Germany] and posterior teeth [LIVDENT GRACE, GC Co., Tokyo, Japan]).

### Follow-up and supportive care protocol

Supportive follow-up therapy was performed every 3 − 6 month, during which clinical parameters (bleeding on probing, suppuration, and peri-implant mucosal redness and swelling) were evaluated, and detailed oral hygiene instructions were provided to the patients. From the perspective of radiation exposure, CBCT imaging was not performed routinely and was conducted only when deemed necessary. When clinical symptoms of sinusitis were observed, CBCT imaging was always performed to establish a diagnosis of sinusitis.

### Clinical outcomes

#### Implant failure

Implant failure was defined as the removal of an implant owing to symptoms such as implant mobility, persistent pain, swelling, inflammation leading to sinusitis and/or peri-implantitis, or oro-antral communication. Patient-level failure was defined as failure when one or more implants were lost in a single patient.

#### Examination of risk factors affecting failure

Risk factors affecting failure were classified as follows:

- Implant-related factors (ZI systems, ZI length, primary stability of the ZI, and position of the ZI’s platform).
- Patient-related factors (sex, smoking history [number of cigarettes: 10 cigarettes/day or more], and systemic diseases [systemic condition which requires regular follow-up at a medical institution such as diabetes, osteoporosis and cardiovascular diseases; excluding localised condition, such as cataracts [e.g.]) Information on smoking history was collected from existing medical records; however, because it was difficult to accurately determine the detailed duration of smoking, pack-year calculations were not performed. Patients who smoked ≥ 10 cigarettes per day were classified as smokers [16]. Systemic diseases could not be classified in detail because the severity of the diseases and medication status changed over the long follow-up period.The patients were those with diabetes mellitus who were receiving treatment to ensure adequate diabetic control, with HbA1c levels maintained below 6.9%.
- Multivariate analysis of the factors affecting the implant survival rate.

### Statistical analysis

Cumulative survival rates were estimated using the Kaplan–Meier method. Implant failure was defined as the event of interest, and implants or patients without failure at the final follow-up were treated as censored observations. Numbers at risk were presented at each time point in the Kaplan–Meier survival curves.

Differences in survival distributions between groups were evaluated using the log-rank test. For variables with more than two categories, an overall log-rank test was first performed. When appropriate, pairwise log-rank comparisons were subsequently performed with Bonferroni correction for multiple comparisons.

Risk factors for zygomatic implant failure were analysed separately at the implant and patient levels. Implant-level variables included implant system, implant length, primary stability, and platform position. Patient-level variables included sex, smoking status, and systemic disease. Univariate survival analyses were first performed for each variable.

To evaluate factors associated with implant failure while accounting for follow-up duration and censoring, Cox proportional hazards regression analyses were performed. Hazard ratios (HRs) and 95% confidence intervals (CIs) were calculated. Multivariable Cox proportional hazards models were constructed using implant-related variables for implant-level analysis and patient-related variables for patient-level analysis. Because the number of failure events was limited, the multivariable Cox analyses were considered exploratory and interpreted cautiously.

The proportional hazards assumption was assessed for the Cox models. Statistical analyses were performed using IBM SPSS Statistics 20 for Windows (IBM Corp., Armonk, NY, USA) and R software, using RStudio. A two-sided p-value of < 0.05 was considered statistically significant.

## Results

Patient and ZI characteristics are shown in Tables 1 and 2. In total, 203 patients were included, with 323 ZIs and 600 CIs being implanted. There were 125 cases in which a total of four implants (ZIs and CIs) were placed; 46 cases in which one additional CI was placed; 31 cases in which two additional CIs were placed; and one case in which three additional CIs were placed. The study included 108 male and 95 female patients, with a mean age of 54.4 ± 9.5 years at the time of surgery. The mean observation period was 99.4 ± 34.1 months.

**Table 1:** Descriptive patient data

	Number
Total	203
Sex	
Male	108
Female	95
Age (years; mean ± SD)	54.4 ± 9.5
Observation period (months; mean ± SD)	99.4 ± 34.1
Smoker	
Yes	78
No	125
Systemic disease	
Healthy	128
Diabetes	5
Osteoporosis	1
Circulatory diseases	20
Combination of diabetes and circulatory diseases	5
Other^†^	44

*SD* standard deviation

^†^For example, dyslipidaemia, breast cancer, thyroid disease

**Table 2:** Descriptive implants data

Implants information		Number
Conventional implants		600
Zygomatic implants		323
Zygomatic implant systems	Brånemark System Zygoma TiUnite	65
	NobelZygoma 45°	114
	NobelZygoma 0°	144
Zygomatic implant length (mm)	35	11
	37.5	4
	40	67
	42.5	48
	45	105
	47.5	31
	50	47
	52.5	10
Primary stability of zygomatic implant (Nཥcm)	< 35 Ncm	5
	≥ 35 Ncm and < 50 Ncm	23
	≥ 50 Ncm	295
Position of the zygomatic implants’ platform	Palatal	82
	Ridge	97
	Vestibular	144

*SD* standard deviation

^†^For example, dyslipidaemia, breast cancer, and thyroid disease

### Cumulative implant survival rate

In total, 11 ZIs and 7 CIs were lost. The number of patients with lost implants was 9 and 4 for ZIs and CIs, respectively. Two ZIs were lost in 2 patients, 3 CIs were lost in 1 patient, and 2 CIs were lost in 1 patient. One patient lost 1 ZI and 1 CI. In total, 4 ZIs (in 4 patients) and 2 CIs (in 2 patients) were lost within 12 months.

**Fig. 3:**
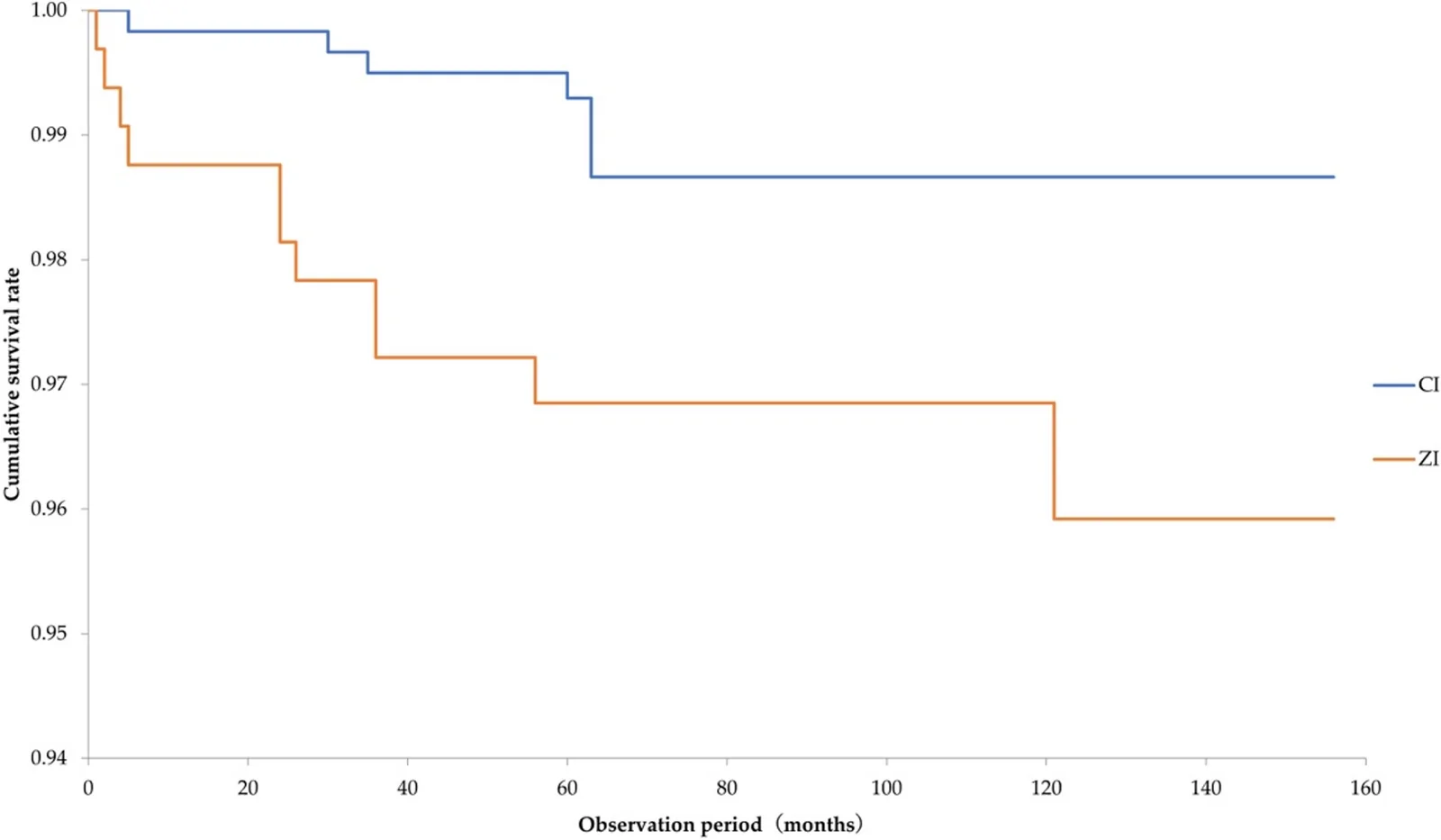
Kaplan–Meier curves at the implant level. The log-rank test demonstrated a significant difference between zygomatic implants (ZIs) and conventional implants (CIs) (*p* < 0.05)

**Fig. 4:**
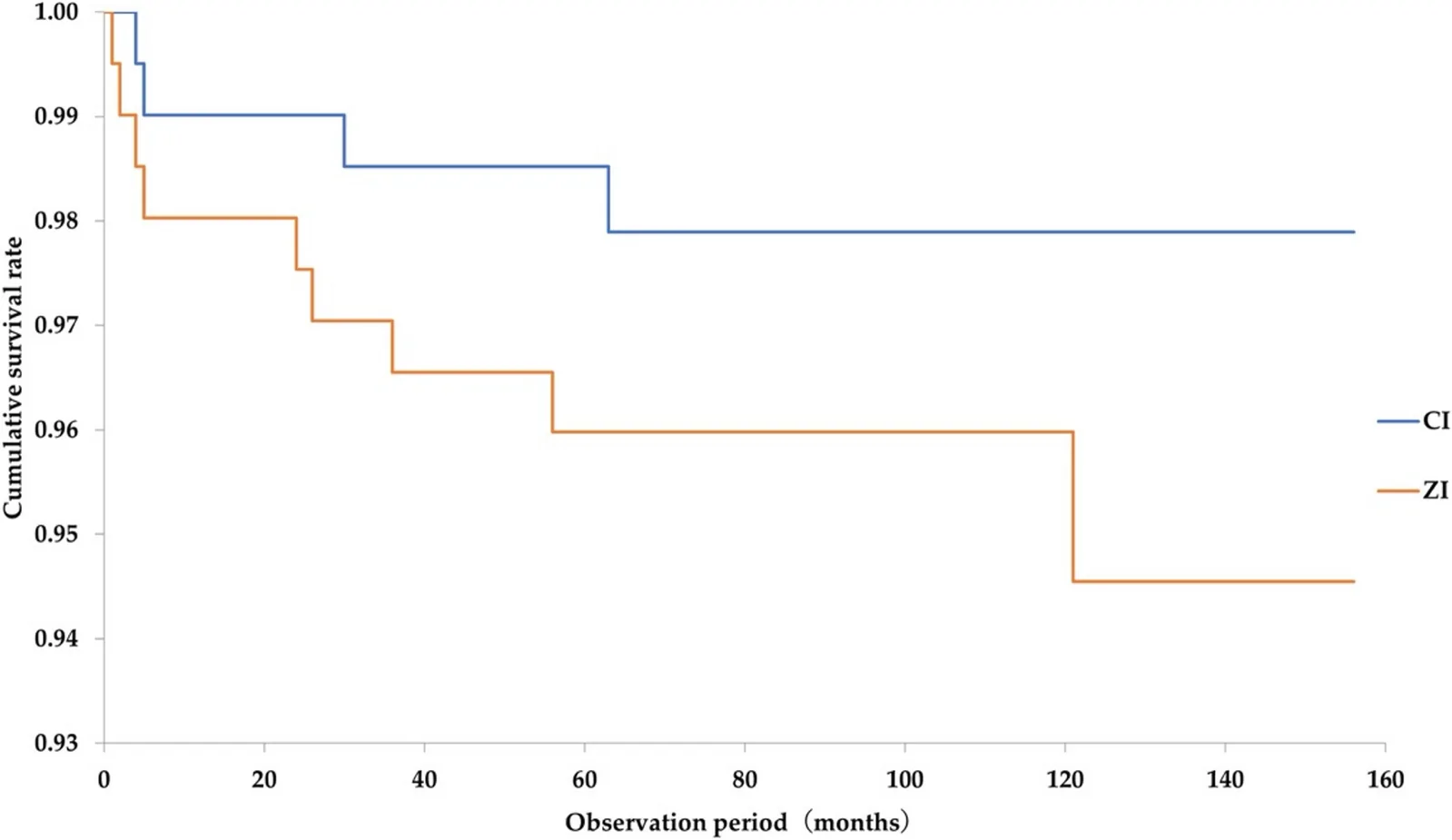
Kaplan–Meier curves at the patient-level implant survival rate. The log-rank test showed no significant difference between zygomatic implants (ZIs) and conventional implants (CIs) (*p* = 0.1598)

The cumulative implant survival rate for ZIs over 3 − 13 years was 94.5% and 95.9% at the patient and implant level, respectively. For CIs, the cumulative survival rates were 97.9% and 98.7% at the patient and implant level, respectively. The cumulative survival rate for ZIs was significantly lower than that for CIs at the implant level (*p* = 0.0178), but not significantly different at the patient level (Figs. 3 and 4).

### Patient profile for ZI failure

The details for cases of ZI failure are presented in Table 3.

**Table 3:** Implant failure cases

Case	Age	Sex	Smoking	Systemic disease	Type of implant	Length (mm)	Insertion torque of zygomatic implants (Ncm)	Position of the zygomatic implant’s platform	Reason for removal	Time to implant failure (months)
1	58	F	-	Cervical cancer	Nobel Zygoma 0°	40	≥ 50	Vestibular	Oro-antral communication	24
					Nobel Zygoma 0°	40	≥ 50	Vestibular	Oro-antral communication	24
2	74	F	་	Dyslipidaemia	Nobel Zygoma 0°	45	≥ 50	Ridge	Sinusitis	121
3	56	M	་	Dyslipidaemia	Brånemark System Zygoma TiUnite	40	≥ 50	Ridge	Sinusitis	56
4	44	M	+	-	Nobel Zygoma 0°	50	≥ 50	Palatal	Sinusitis	2
5	52	M	-	Diabetes, Hypertension	Nobel Zygoma 45°	45	≥ 50	Ridge	Sinusitis	36
					Nobel Zygoma 45°	45	≥ 50	Palatal	Sinusitis	36
6	55	M	-	Dyslipidaemia	Nobel Zygoma 45°	47.5	≥ 50	Palatal	Peri-implantitis	26
7	49	M	་	Hypothyroidism	Nobel Zygoma 45°	50	≥ 50	Palatal	Sinusitis	4
8	61	M	་	Hypertension, Dyslipidaemia, Hyperuricaemia	Nobel Zygoma 45°	50	35	Palatal	Peri-implantitis	5
9	60	M	་	Myocardial infarction, Hypertension, Dyslipidaemia	Nobel Zygoma 45°	50	≥ 50	Palatal	Alveolar bone fracture	1

### Risk factors related to the survival rate

#### Implant-related factors

##### ZI type

When the differences in survival rate according to the ZI type were considered, Brånemark System Zygoma TiUnite (Nobel Biocare AG, Kloten, Switzerland), NobelZygoma 45° (Nobel Biocare AG, Kloten, Switzerland), and Nobelzygoma 0° exhibited survival rates of 98.5%, 94.7%, and 97.2%, respectively (Table 4). No significant differences were observed among the survival rates according to the ZI type.

**Table 4:** Type of zygomatic implant and survival rate

Zygomatic implant type	Number of implants placed	Number of implant failure cases	Survival rate (%)	*p*-value
Brånemark System Zygoma TiUnite	65	1	98.5%	0.100
NobelZygoma 45°	114	6	94.7%	
NobelZygoma 0°	144	4	97.2%	

*p < 0.05** *p* < 0.01: Log-rank test

##### ZI length

Regarding the differences in survival rate according to the ZI length, implants of < 40 mm in length exhibited survival rates of 100%; those between 40 mm and 45 mm exhibited survival rates of 97.4%, those between 45 mm and 50 mm exhibited survival rates of 99.3%; and those ≥ 50 mm exhibited survival rates of 93.0% (Table 5). No significant differences in survival rates were observed according to the ZI length.

**Table 5:** Zygomatic implant length and survival rate

Zygomatic implant length	Number of implants placed	Number of implant failure cases	Survival Rate (%)	*p*-value
< 40 mm	15	0	100%	0.886
≥ 40 mm and < 45 mm	115	3	97.4%	
≥ 45 mm and < 50 mm	136	4	97.1%	
≥ 50 mm	57	4	93.0%	

*p < 0.05** *p* < 0.01: Log-rank test

##### Primary stability of ZIs

The survival rates according to primary stability at the time of implant placement were as follows: ZIs with a value of < 35 Ncm exhibited survival rates of 100%; those with a value of 35 − 50 Ncm exhibited survival rates of 95.7%; and those with a value of > 50 Ncm exhibited survival rates of 96.6%. No significant differences were observed in the survival rates according to primary stability (Table 6).

**Table 6:** Analysis of primary stability and survival rate

Primary stability of the zygomatic implant	Number of implants placed	Number of implant failure cases	Survival rate (%)	*p*-value
< 35 Ncm	5	0	100%	0.186
≥ 35 Ncm and < 50 Ncm	23	1	95.7%	
≥ 50 Ncm	295	10	96.6%	

*p < 0.05** *p* < 0.01: Log-rank test

##### Position of the ZI platform

The survival rate of the ZI platform when positioned in the vestibular area was 98.6%, whereas it was 96.9% when positioned on the ridge and 92.7% when positioned in the palatal region (Table 7). The survival rate of the platform in the palatal region was significantly lower (*p* = 0.003).

**Table 7:** Platform position of zygomatic implant and survival rate

Platform position	Number of implants placed	Number of implant failure cases	Survival rate (%)	*p*-value
Vestibular region	144	2	98.6%	0.003**
On the ridge	97	3	96.9%	
Palatal aspect	82	6	92.5%	

*p < 0.05** *p* < 0.01: Log-rank test

#### Patient-related factors

##### Sex

For ZIs, male patients exhibited survival rates of 93.5% at the patient level and 95.4% at the implant level. Female patients exhibited survival rates of 97.9% at the patient level and 98.0% at the implant level, with no significant difference between the sexes (Table 8).

**Table 8:** Patient sex and survival rate

Zygomatic implant	Number of patients	Number of implants	Patient level (number of patient failure cases)	*p*-value	Implant level (number of implant failure cases)	*p*-value
Male	108	175	93.5% (7)	0.202	95.4% (8)	0.096
Female	95	148	97.9% (2)		98.0% (3)	

*p < 0.05** *p* < 0.01: Log-rank test

##### Smoking status

Smokers exhibited implant survival rates of 92.3% at the patient level and 95.3% at the implant level. Non-smokers exhibited implant survival rates of 97.6% at the patient level and 97.4% at the implant level (Table 9). Low survival rates at both patient and implant levels were observed in smokers; however, no significant differences were found (*p* = 0.023).

**Table 9:** Smoking and survival rate

Zygomatic implant	Number of patients	Number of implants placed	Patient level (number of patient failure cases)	*p*-value	Implant level (number of implant failure cases)	*p*-value
Smoker	78	127	92.3% (6)	0.037*	95.3% (6)	0.211
Non smoker	125	196	97.6% (3)		97.4% (5)	

*p < 0.05** *p* < 0.01: Log-rank test

##### Systemic disease

In this study, we investigated the association of the survival rate of implants according to systemic disease. Furthermore, diabetes mellitus [17, 18], cardiovascular disease [18–20], and osteoporosis [21], which have been reported as causes of implant failure and peri-implantitis, were investigated individually.

For ZIs, healthy participants exhibited survival rates of 99.2% at the patient level and 99.5% at the implant level. Patients with systemic disease exhibited survival rates of 89.3% and 91.6% at the patient and implant level, respectively (Table 10). The survival rate was significantly low for patients with systemic disease at both patient and implant levels (patient level, *p* = 0.002; implant level, *p* = 0.000).

**Table 10:** Systemic disease and survival rate

Maxilla	Number of patients	Number of implants placed	Patient level (number of patient failure)	*p*-value	Implant level (number of implant failure)	*p*-value
Systemic disease						
Absence	128	204	99.2% (1)	0.0032**	99.5% (1)	0.000**
Presence	75	119	89.3% (8)		91.6% (10)	
Diabetes	5	7	100% (0)		100% (0)	
Osteoporosis	1	2	100% (0)		100% (0)	
Circulatory diseases	20	29	90.0% (2)		93.1% (2)	
Combination of diabetes and circulatory diseases	5	9	80.0% (1)		77.8% (2)	
Others	44	72	88.6% (5)		91.7% (6)	

*p < 0.05** *p* < 0.01: Log-rank test

#### Multivariable cox proportional hazards analysis of factors associated with implant survival (Table 11)

**Table 11:** Multivariate analysis of the factors affecting the implant survival

Implant-related factors for implant-level	Risk factor	Hazard ratio	*p*-value
Platform position of the zygomatic implant	Palatal	18.177 [1.418–233.053]	0.026 *
	Ridge	6.054 [0.533–68.784]	0.146
Patient-related factors for patient-level			
Systemic disease	Presence	14.872 [1.812–122.077]	0.012 < 0.05*

The 95% confidence interval is displayed in brackets

*p<0.05: Cox Proportional Hazards regression

To identify factors associated with decreased survival while accounting for follow-up duration and censoring, Cox proportional hazards regression analyses were performed. Variables that showed significant associations in the univariate log-rank analyses, as well as clinically relevant variables, were included in the multivariable models.

In the patient-level Cox proportional hazards model including sex, smoking status, and systemic disease, systemic disease remained significantly associated with reduced survival (HR = 14.872, 95% CI: 1.812-122.077, *p* = 0.012). Smoking status was significant in the univariate log-rank analysis but did not remain statistically significant after adjustment.

In the implant-level Cox proportional hazards model including implant-related variables, palatal positioning of the ZI platform was significantly associated with reduced implant survival (HR = 18.177, 95% CI: 1.418–233.053, *p* = 0.026). Because the number of failure events was limited, these multivariable Cox analyses were considered exploratory and were interpreted cautiously.

## Discussion

A systematic review of the survival rates for implant-supported fixed prosthodontic treatment for fully edentulous maxilla [22] reported rates of 94.95 to 100% for the delayed loading protocol (follow-up period: 2–15 years), 94.7 to 100% for the early loading protocol (follow-up period: 1–3 years), and 90.43 to 100% in the maxilla for the immediate loading protocol (follow-up period: 1–10 years).

The implant survival rate for treatment based on the all-on-four concept in the maxilla using CIs only is reportedly 93.9% to 100% (follow-up period: 3–18 years) [23]. In our study, the ZI cumulative survival rate with a follow-up period of 3–13 years was 94.5% and 95.9% at the patient and implant level, respectively. The CI cumulative survival rate was 97.9% and 98.7% at the patient and implant level, respectively. Compared to previous studies [10–12, 22, 23], this suggests that treatment based on the all-on-four concept combining ZIs could be beneficial, even for Japanese populations.

Although ZIs were initially used in conjunction with delayed loading, with subsequent improvement in the surgical techniques, implant design, and surface shape, ZIs have been used for treatment with immediate loading [7, 8, 24]. According to a systematic review by Brennand Roper et al. [10], the ZI survival rates with loading protocol were higher for immediate loading (98.1% [95% CI: 96.2–99.0], mean follow-up period: 73.6 months) than for delayed loading (95% [95% Cl: 91.7–97.1], mean follow-up period: 69.3 months). A systematic review [25] also revealed a similar trend, which may be attributed to improvements in the ZI surface shape and surgical techniques [10]. Moreover, achieving primary stability is a crucial factor for immediate loading protocols. In the present study, no significant difference in survival rates was observed according to primary stability. However, because ZIs and CIs differ in their anchorage sites (zygomatic versus alveolar bone) and biomechanical environments, it is difficult to determine whether the insertion torque thresholds established for immediate loading of CIs can be directly applied to ZIs. Therefore, further investigation is required to define appropriate insertion torque values for immediate loading of ZIs. While the advantages of ZIs—including avoiding bone augmentation and the possibility of immediate loading treatment—have been well recognised, their placement is technically challenging and the success or failure of treatment depends greatly on the skills and experience of the surgeon. Therefore, specialised knowledge of surgery and recovery is necessary to address the potential complications following implant placement [9]. In recent years, dynamic computer-aided surgery (d-CAIS) has advanced and has been applied to ZI placement, with clinically acceptable accuracy reported in a systematic review [26]. However, when the accuracy of ZI placement using d-CAIS was compared between trans-sinus and extra-sinus approaches, lower accuracy was reported for ZIs placed using the extra-sinus approach [27]. If an extra-sinus ZI deviates even slightly from its intended position and loses contact with the maxillary bone, maxillary support is compromised, resulting in a marked increase in stress not only on the zygomatic bone but also on the implant itself under functional loading, which in turn may lead to significant mechanical and biological complications [28]. Because correction of the position of a deviated ZI is also difficult, careful consideration and meticulous execution are required.

In this study, comparison of the cumulative CI and ZI survival rates indicated that the survival rate was significantly lower for ZIs (95.9%) than for CIs (97.9%) at the implant level (*p* = 0.0179).

Moraschini et al. [29] reported survival rates of 96.5% ± 5.02% and 95.8% ± 6.36% for ZIs and CIs (mean follow-up period: 78 months), respectively, while Pesce et al. [30] reported survival rates of 97% and 95.7% for ZIs and CIs (mean follow-up period: 36 months), respectively for treatments combining ZIs and CIs. Thus, in contrast to our results, neither of the above reports demonstrated a significant difference, which could be attributed to the differences in the follow-up periods. Brennand Roper et al. [10] reported in a systematic review that although the total annual ZI loss rate was 0.7%/year (95% CI: 0.4–1.0), the ZI loss rate within 1 year after implantation was 2% (95% CI: 1.1–3.7). From the second year, the ZI loss rate was 0.5%/year (95% CI: 0.3–0.7), indicating that the loss rate was higher in the first year after implantation. Chrcanovic et al. [25] reported a 12-year cumulative survival rate of 95.21% in a systematic review, with most losses being detected within 6 months postoperatively and ZI losses occurring in the early postoperative period. However, they also stated that since very few studies have followed up patients for 5 years or longer, the results should be interpreted with caution. The mean follow-up period in the present study was 99.4 ± 34.1 months, which was longer than that of previous studies comparing the ZI and CI survival rates [29, 30]. One case of ZI loss due to paranasal sinusitis was observed at 121 months postoperatively.

Paranasal sinusitis was the most common cause of ZI loss. In their systematic review, Brennand Roper et al. [10] noted that although the prevalence of paranasal sinusitis accompanying ZI implantation was 14.2% (95% CI: 8.8–22.0, mean follow-up period: 65.4 months), the diagnosing methods for paranasal sinusitis varied considerably among studies, including the assessment of clinical symptoms, radiographic images, patient questionnaires, or a combination of all three. Meanwhile, in their systematic review, Chrcanovic et al. [25] described a low prevalence of paranasal sinusitis (2.4%). They noted that as many studies did not describe the prevalence of comorbidities, the prevalence of paranasal sinusitis may have been underestimated. In the present study, postoperative CBCT imaging was not performed routinely, considering radiation exposure associated with imaging, and was conducted only when clinical symptoms of sinusitis were present. Consequently, asymptomatic sinusitis may not have been detected. Di Cosola et al. [14] reported a 36.4% prevalence of paranasal sinusitis (95% CI: 20.4–54.9, mean follow-up period: 141.6 months). Bothur et al. [31] reported that radiographic evaluation by CBCT for long-term follow-up (mean follow-up period: 9.3 years) of ZIs placed into the maxillary sinus revealed high prevalence of maxillitis attributed to chronic rhinosinusitis in the posterior wall of the maxillary sinus, which does not directly impact surgery (26 out of 27 maxillary sinuses) and closure of the maxillary ostium in the ethmoid infundibulum (9 out of 27 maxillary sinuses), which may have contributed to the increased prevalence associated with longer follow-up periods.

The mechanism for ZI implantation attributed to paranasal sinusitis could be a lack of osseous union (contact) between the palatal bone and ZI, and ZI movement in the crown long axis direction while creating a communication between the maxillary sinus and oral cavity [10, 31], thereby increasing the risk of paranasal sinusitis due to exposure of the ZI within the paranasal cavity [10, 24, 31–33]. Scattered reports on the prevalence of paranasal sinusitis associated with the original surgical technique (OST)—involving implant placement from the palatal side through the maxillary sinus—and the AGA—involving implantation outside the maxillary sinus—have suggested a higher prevalence with the OST [8, 10, 24, 29, 32, 33].

In the present study, the log-rank analysis showed that ZIs with a palatal platform position had significantly lower survival than those with other platform positions. Furthermore, in the implant-level Cox proportional hazards model, palatal platform positioning remained significantly associated with reduced ZI survival (HR = 18.177, 95% CI: 1.418–233.053, *p* = 0.026). The most common cause of ZI loss was paranasal sinusitis, accounting for 6 of 11 failed implants (54.5%). These findings suggest that palatal platform positioning may be related to an increased risk of biologic complications, including paranasal sinusitis. However, because the number of failure events was limited and the confidence interval was wide, this association should be interpreted cautiously.

Meanwhile, Kämmerer et al. [33] reported in a systematic review that ZI survival rates ranged from 90.3% to 100% for the original surgical technique and from 90.4% to 100% for the anatomy-guided approach, with broadly comparable clinical outcomes between the techniques.

The only radiographic findings were thickening of the paranasal sinus mucosa, with no clinical symptoms [8, 24]. Most cases of paranasal sinusitis improved with antibiotic administration or curettage [10]. Moreover, thickening of the paransal sinus mucosa only was not related to ZI loss [8, 24, 34, 35]. Based on these scattered reports, further research on the association between the severity of paranasal sinusitis and ZI prognosis is warranted.

In the present study, no sex-related significant differences were observed in the implant survival rates. However, Di Cosola et al. [14] reported that the risk of ZI loss was higher in male individuals. In their investigation of treatment based on the all-on-four concept in the maxillary jaw using CIs only, Maló et al. [36] found low CI survival rates and increased marginal bone absorption in male individuals, warranting careful follow-up.

In our study, systemic disease exhibited the greatest impact on ZI survival rates. Because a significant difference in the cumulative implant survival rate was observed at the patient level, it is plausible that age-related changes in systemic conditions, including the severity of systemic diseases, may have influenced outcomes.

Although no studies have reported on the impact of systemic disease on ZI survival rates, Niedermaier et al. [37] reported systemic disease as a risk factor for CI loss in patients treated using the all-on-four concept using only CIs. The risks of peri-implantitis and implant failure are increased in patients with poorly controlled diabetes [17, 18] and cardiovascular disease [18–20], and patients using bisphosphonates to treat osteoporosis [21]. However, as D’Ambrosio et al. [38] reported that systemic diseases, such as mental illness, HIV infection, hypothyroidism, and cardiovascular disease do not affect CI survival rates, further investigation through additional research is warranted. Other reports have indicated that systemic disease increases marginal bone loss (MBL) around the CI [37, 38], with Carlos et al. [34] reporting that the MBL onset rates were high in patients with hypertension and hyperlipidaemia. During ZI insertion, if the ZI platform position is located on the palatal region or on the ridge, even a slight increase in MBL may lead to insufficient osseous union (contact) between the bone surrounding the platform and the ZI, resulting in increased risk of fistula formation between the maxillary sinus and oral cavity, and subsequent paranasal sinusitis. However, subtle MBL occurring around the implant platform is difficult to detect on dental panoramic radiographs because of the superimposition of surrounding structures and to detect on CBCT images owing to imaging artifacts. Consequently, MBL is often identified only after it has progressed or when inflammation has extended to the surrounding soft tissues, highlighting the need to establish methods for early detection.

In the present study, no significant differences in survival rates were observed among the ZI systems. However, finite element analyses have demonstrated stress concentration around the ZI platform [28, 39]. Under such conditions, the progression of MBL may occur and promote an increased bending moment. Therefore, the presence or absence of threads that help distribute stress may influence implant survival rates.

Furthermore, with increase in the follow-up periods, aging of patients is generally associated with higher prevalence of systemic disease. As the onset rates of paranasal sinusitis may increase with longer follow-up rates [14, 31], more careful follow-up is warranted due to the risk of treatment that includes the use of ZIs resulting in long-term complications.

The results of this study clarified the palatal region of the ZI platform as a specific risk factor influencing the survival rate of ZIs, and suggested a link with sinusitis. Furthermore, whilst the presence or absence of systemic disease had the greatest impact on the survival rate of ZIs, the exact reason for this remains unclear, and it was considered that further research is required to investigate them.

This study has some limitations. First, it was conducted at a single institution. Second, the retrospective study design, which relied on data collected from existing medical records, may be subject to incompleteness and inconsistencies in the records, thereby limiting control over potential confounding factors. Third, the age range of the study population was relatively narrow (54.4 ± 9.5 years), and relatively few elderly patients—who are a primary target population for ZI treatment—were included; this factor should also be taken into consideration. Fourth, although Cox proportional hazards regression analyses were performed to account for follow-up duration and censoring, the number of ZI failure events was limited, with only 11 failed ZIs observed during the study period. Therefore, the multivariable Cox models may have been statistically underpowered, and the hazard ratios, particularly those with wide confidence intervals, should be interpreted cautiously. These analyses should be regarded as exploratory rather than definitive evidence of independent risk factors. Fifth, the scope of this study was limited because mechanical complications of prosthetic devices were not investigated. In this study, no framework fractures were observed in any of the cases; however, the existing medical records did not contain sufficient information regarding screw loosening or screw fracture, which prevented a comprehensive analysis of these complications. Conventionally, screw-retained prostheses are considered to be prone to screw and abutment loosening. Moreover, ZI bending movements cause further vibration of the prostheses, which is thought to make them even more prone to loosening [31, 40, 41]. Variations in peri-implant bone conditions associated with different ZI placement techniques can also influence the stress and displacement acting on ZIs [39]. Additionally, the deviation of the platform position from the ridge toward palatal or vestibular locations has been associated with a higher incidence of prosthesis-related complications [42]. Thus, additional data are needed to assess the impact of the bending movements of ZIs.

## Conclusions

We investigated the 3–13 year cumulative survival rates of implants used in treatment based on the all-on-four concept combined with ZIs and examined the risk factors that influenced survival rates. The following results were obtained:

1. Although both ZIs and CIs had high survival rates, they were significantly lower for ZIs than for CIs at the implant level.
2. Investigation of the implant-related factors revealed that the survival rate when ZI platform was positioned in the palatal region was significantly lower, and a correlation with paranasal sinusitis was suggested.
3. Investigation of the patient-related factors revealed that the presence of systemic disease significantly reduced the survival rates. However, the impact of systemic disease on the ZI survival rates is questionable.
4. Although we were able to confirm that treatment based on the all-on-four concept combining ZIs in Japanese patients resulted in a favourable prognosis even in the long-term, our results suggested that more careful follow-up is required due to the risk of increased number of complications such as paranasal sinusitis with longer follow-up periods.

## Data Availability

The raw data supporting the conclusions of this article will be made available by the corresponding author on request.

## Abbreviations

AGA anatomy: Guided approach
CI: Conventional implant
MBL: Marginal bone loss
OST: Original surgical technique
ZI: Zygomatic implant
